# The impact of infliximab treatment on quality of life in patients with inflammatory rheumatic diseases

**DOI:** 10.1186/ar2306

**Published:** 2007-10-08

**Authors:** Chenglong Han, Josef S Smolen, Arthur Kavanaugh, Désirée van der Heijde, Jürgen Braun, René Westhovens, Ning Zhao, Mahboob U Rahman, Daniel Baker, Mohan Bala

**Affiliations:** 1 Centocor Research and Development, Inc., 200 Great Valley Parkway, Malvern, Pennsylvania, 19355 USA; 2 Division of Rheumatology, Internal Medicine III, Medical University of Vienna and Hietzing Hospital, Waehringer Guertel 18-20, A-1090, Vienna, Austria; 3 Division of Rheumatology, Allergy, and Immunology, University of California, San Diego, 9500 Gilman Drive, LaJolla, California, 92093 USA; 4 Department of Rheumatology, Leiden University Medical Center, PO Box 9600, 2300 RC Leiden, The Netherlands; 5 Rheumazentrum Ruhrgebiet, Landgrafenstrasse 15, D-44652 Herne, Germany; 6 Division of Rheumatology, UZ Gasthuisberg, Herestraat 49, 3000 Leuven, Belgium

## Abstract

In this study, we compare the health-related quality of life (HRQoL) of patients with moderate-to-severe rheumatoid arthritis (RA), psoriatic arthritis (PsA), and ankylosing spondylitis (AS), and study the effect of treatment with infliximab on the HRQoL of patients with these diseases. Short Form Health Survey-36 (SF-36) data from the placebo-controlled phases of 4 studies of infliximab in patients with inflammatory rheumatic diseases (*n *= 1990) were evaluated. Data came from the Anti-TNF Trial in Rheumatoid Arthritis with Concomitant Therapy (ATTRACT) (*n *= 428), the Safety Trial for Rheumatoid Arthritis with REMICADE Therapy (START) (*n *= 1083), the Ankylosing Spondylitis Study for the Evaluation of Recombinant Infliximab Therapy (ASSERT) (*n *= 279), and the Infliximab Multinational Psoriatic Arthritis Clinical Trial II (IMPACT II) (*n *= 200). SF-36 assessments were made at weeks 0, 10, 30, and 54 in ATTRACT, weeks 0, 6, and 22 in START, weeks 0, 12, and 24 in ASSERT, and weeks 0 and 14 in IMPACT II. All patient populations had significantly impaired physical aspects of HRQoL at baseline relative to the general population of the United States, and the magnitude of impairment was similar across the diseases. Mean baseline physical component summary scores were 29 in the RA cohort, 32 in the PsA cohort, and 29 in the AS cohort. In all 3 diseases, patients who received infliximab showed significant improvement in physical component summary scores compared with those who received placebo. The magnitude of the difference of improvement (effect size, 95%CI) between infliximab and placebo groups was similar in the AS (10.1, 9.2–11.0), PsA (8.6, 7.8–9.4), and RA (10.1, 9.2–11.0) cohorts. Patients with RA and those with PsA treated with infliximab also showed greater improvement in the mental component summary score than those in the placebo group with an effect size of 4.6 (4.2–5.1) in RA and 2.7 (2.4–3.1) in PsA. Patients in large randomized controlled studies of infliximab in RA, PsA, and AS had similar impairment in physical aspects of HRQoL at baseline and showed significantly greater improvement in HRQoL after treatment with infliximab.

## Introduction

Rheumatoid arthritis (RA), psoriatic arthritis (PsA) and ankylosing spondylitis (AS) are common inflammatory rheumatic diseases with severe consequences for patients' health-related quality of life (HRQoL). RA is the most common of the three, and is characterized by chronic, symmetric, and erosive synovitis of peripheral joints. RA is two to three times more prevalent in women than men and has a peak onset between the ages of 40 and 50 years. Compared with RA, PsA also has articular manifestations, but the joint patterns can be different, including the number of affected joints and the type of arthritis [1]. PsA typically includes psoriatic skin lesions and has a similar prevalence in men and women. Patients with PsA are also usually affected at a younger age than those with RA. Both RA and PsA can lead to impaired function and reduced mobility. AS predominantly affects the axial skeleton and sacroiliac joints (sacroiliitis), which typically leads to decreased spinal mobility. However, AS may also include extraspinal manifestations, such as peripheral arthritis and uveitis, and it is often associated with inflammatory bowel disease and psoriasis. In contrast to RA, AS is three times more prevalent in men than women, and symptoms usually begin in adolescence.

Daily pain, stiffness, fatigue, and physical disability are common features of all three rheumatic diseases. Persistent active disease without effective treatment may lead to permanent loss of physical function, reduced productivity, and increased rates of work disability about 10 years after disease onset [2]. Impairments in physical functioning and disability, and the strategies patients use to cope with them, can significantly affect HRQoL. Traditionally, RA is considered to be the most severe of the three diseases, exerting the greatest impact on physical aspects of HRQoL. However, the cohorts of previous studies that compared the HRQoL of patients with RA to that of patients with PsA or AS consisted primarily of patients with mild-to-moderate disease [3,4]. Disease severity is an important factor influencing the HRQoL of patients. Therefore, the objectives of this study were to compare the HRQoL of patients with moderate-to-severe RA, PsA, or AS, and to compare the effect of treatment with infliximab on the HRQoL of patients with these diseases.

## Materials and methods

This analysis included 1,987 patients from four clinical trials in which the safety and efficacy of infliximab was evaluated in rheumatic diseases. Two of the trials were conducted in patients with RA: the Anti-TNF Trial in Rheumatoid Arthritis with Concomitant Therapy (ATTRACT) (*n *= 428) [5-7] and the Safety Trial for Rheumatoid Arthritis with REMICADE Therapy (START) (*n *= 1083) [8]. The disease severity entry criteria for these studies were similar. All patients must have had active RA with 6 or more tender and swollen joints. In ATTRACT, patients must have also had two or more of the following: morning stiffness ≥45 minutes, erythrocyte sedimentation rate >28 mm/hour, and C-reactive protein >2 mg/dL. Patients in both studies must have demonstrated these symptoms despite receiving stable doses of MTX (≥12.5 mg/week for patients in ATTRACT). Patients in ATTRACT were not permitted to have significant comorbidities or concomitant medications, whereas patients were permitted in START as long as they did not have conditions or concomitant medications that were prohibited by the product labeling.

Data from the Ankylosing Spondylitis Study for the Evaluation of Recombinant Infliximab Therapy (ASSERT) (*n *= 279) were used to assess HRQoL in AS [9], and data from the Infliximab Multinational Psoriatic Arthritis Clinical Trial II (IMPACT II) (*n *= 200) [1] were used to assess HRQoL in PsA. Patients were eligible for the ASSERT study if they had a Bath Ankylosing Spondylitis Disease Activity Index score of ≥4 (range 0 to 10) and a spinal pain assessment score of ≥4 on a visual analog scale (range 0 to 10). Patients were eligible for the IMPACT II study if they had ≥5 tender and ≥5 swollen joints and either CRP levels of ≥15 mg/L and/or morning stiffness lasting ≥45 minutes.

All studies were conducted at multiple study sites in North America and Europe. The START study was also conducted at sites in South America.

Detailed descriptions of patient selection criteria and study designs for these trials have been published elsewhere [5-10]. As indicated in these reports, all studies were conducted in compliance with the Declaration of Helsinki, and all patients gave written informed consent to participate in the study. The impact of treatment on the HRQoL of patients in the ATTRACT [6] and IMPACT II [11] studies have also been published previously. In each study, patients were randomly assigned to receive either infliximab or placebo in a 3-dose induction regimen followed by maintenance therapy. Patients in ATTRACT received infliximab at 3 mg per kg of body weight or 10 mg per kg of body weight every 4 or 8 weeks, patients in START received infliximab at 3 mg per kg body weight or 10 mg per kg body weight every 8 weeks, patients in ASSERT received 5 mg per kg body weight every 6 weeks, and patients in IMPACT II received 5 mg per kg body weight every 8 weeks. All patients in RA clinical trials received concomitant methotrexate (MTX). Patients in IMPACT II were allowed to continue therapy with stable doses of MTX if they were receiving it at baseline. None of the patients in ASSERT received concomitant MTX.

HRQoL was assessed using Short Form Health Survey-36 (SF-36) at weeks 0, 10, 30, and 54 in ATTRACT; weeks 0, 6, and 22 in START; weeks 0, 12, and 24 in ASSERT; and weeks 0 and 14 in IMPACT II. The norm-based SF-36 scoring system was used to calculate physical and mental components summary scores (PCS, MCS) as well as the 8 individual scales. Scores for each scale range from 0 to 100 with higher SF-36 scores being indicative of better HRQoL and a score of 50 ± 10 representing the mean ± standard deviation for the general population of the United States. Baseline SF-36 scores and changes from baseline after treatment were adjusted for age, sex, and disease duration when comparing across diseases using analysis of covariance (ANCOVA). Effect size and 95% confidence interval (CI) of change after treatment between treatment groups was estimated using Hedges' method [12].

## Results

### Baseline demographic characteristics

Demographic characteristics of patients in each study were typical of those with moderate-to-severe disease (Table 1). The average age of patients with RA was 52.1 years. Patients with PsA (46.8 years) and AS (39.8 years) were typically younger than those with RA. Most patients with RA (80%) were women, while 39% of patients with PsA and 19% of patients with AS were women. Most patients had well-established disease, with average disease durations of 10.2 years for patients with AS, 10.0 years for patients with RA, and 7.9 years for patients with PsA.

**Table 1:** Age, sex, and disease duration since diagnosis

	Rheumatoid arthritis^a^		Psoriatic arthritis		Ankylosing spondylitis	
	Placebo *n *= 450	IFX *n *= 1061	Placebo *n *= 100	IFX *n *= 100	Placebo *n *= 78	IFX *n *= 201
Sex, % women	82%	78%	49%	29%	13%	22%
Age (years)^b^	52.2 (12.3)	51.8 (12.4)	46.5 (11.3)	47.1 (12.8)	40.3 (9.4)	39.6 (10.6)
Range	(19, 85)	(18, 89)	(24, 71)	(18, 80)	(20, 74)	(18, 71)
Disease duration (years)^b^	9.9 (8.8)	10.3 (9.1)	7.5 (7.8)	8.4 (7.2)	11.9 (8.0)	10.1 (8.7)
Range	(0.3, 41.5)	(0.3, 55.5)	(0.3, 38.5)	(0.4, 29.2)	(0.3, 31.6)	(0.3, 41.1)

^a^All patients were receiving concomitant methotrexate; ^b^Values are mean (standard deviation) unless otherwise indicated. IFX, infliximab.

### Quality of life at baseline

**Figure 1:**
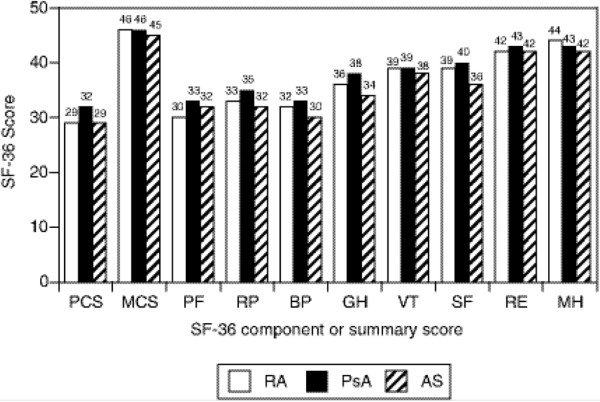
**Health-related quality of life at baseline**. Mean norm-based Short Form Health Survey-36 (SF-36) scales, mental component summary score (MCS), and physical component summary score (PCS) at baseline. Values were adjusted for age, gender, and disease duration. BP, bodily pain; GH, general health; MH, mental health; PF, physical functioning; RE, role emotional; RP, role physical; SF, social functioning; VT, vitality.

**Figure 2:**
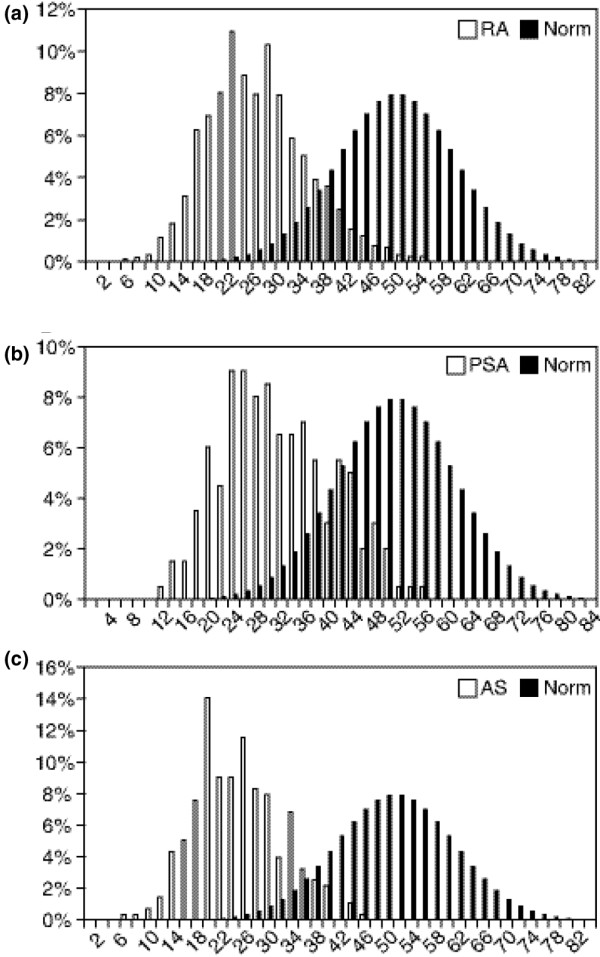
**Frequency distribution of physical component summary scores**. Frequency distribution of baseline physical component summary scores (PCS) for patients with rheumatoid arthritis (RA) (a), psoriatic arthritis (PsA) (b), and ankylosing spondylitis (AS) (c) in infliximab clinical trials.

Overall, after adjustment for age, sex, and disease duration, the physical HRQoL, which includes mean physical functioning, role physical, and bodily pain, was similar for patients with RA, PsA, and AS at baseline and was well below the average score of 50 for the general United States population (Figure 1). Thirty was the lowest mean score for an individual scale and was observed in role physical for patients with RA and bodily pain for patients with AS. However, mean scores for all scales were similar among the three diseases, with no more than 4-point differences between means. The distributions of the baseline PCS scores were shifted left-sided in all three patients groups (Figure 2), and the average PCS scores were about 2 standard deviations lower than general population means, with mean scores of 29 for RA, 32 for PsA, and 29 for AS. The MCS scores were similar in all three patients groups with a mean of 46 for both RA and PsA patients and a mean of 45 for patients with AS.

### Improvement in quality of life after treatment

**Figure 3:**
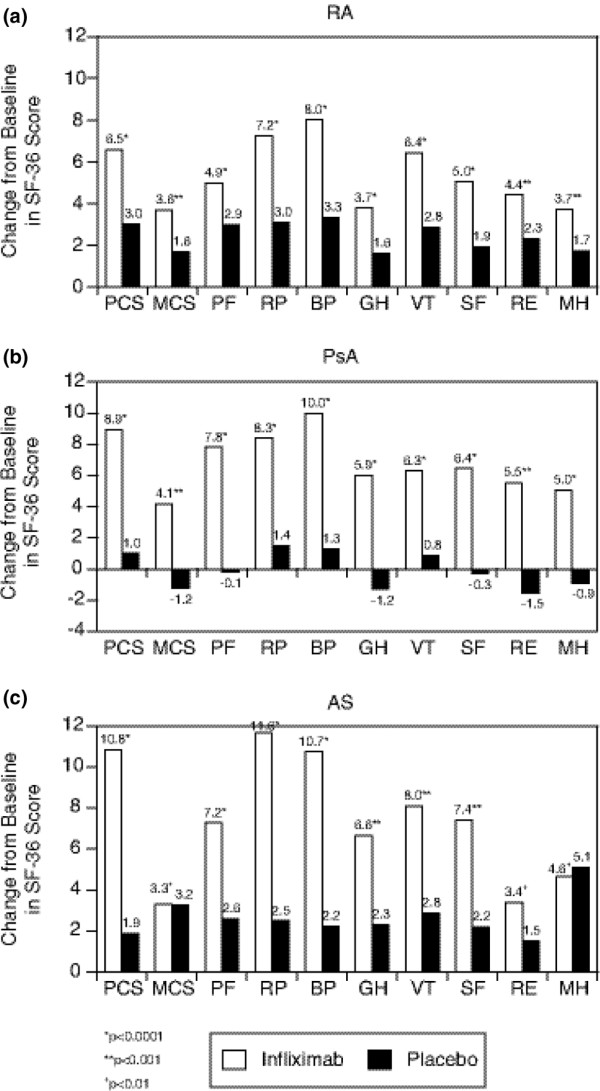
**Change in health-related quality of life after treatment**. Mean changes from baseline to the first assessment time point in Short Form Health Survey-36 (SF-36) scales for patients with rheumatoid arthritis (RA) (a; change from baseline to week 6 or 10), patients with psoriatic arthritis (PsA) (b; change from baseline to week 14), and patients with ankylosing spondylitis (AS) (c; change from baseline to week 12). BP, bodily pain; GH, general health; MCS, mental component summary score; MH, mental health; PCS, physical component summary score; PF, physical functioning; RE, role emotional; RP, role physical; SF, social functioning; VT, vitality.

After treatment with infliximab, patients with RA, PsA, or AS had a significantly greater improvement from baseline to the first assessment time point in all physical scales of the SF-36 compared with patients who received placebo (Figure 3). In each of the three diseases, the greatest improvements from baseline were observed in role physical and bodily pain. Overall, the magnitude of the difference in the improvement (effect size, 95% CI) on PCS between patients treated with infliximab and placebo was similar in the AS (10.1, 9.2–11.0), PsA (8.6, 7.8–9.4), and RA (10.1, 9.2–11.0) cohorts after adjustment for sample size and standard deviation. Patients with RA and PsA treated with infliximab also showed greater improvement in the MCS than placebo. The effect size of infliximab relative to placebo was 4.6 (4.2–5.1) in RA and 2.7 (2.4–3.1) in PsA. In the AS cohort, the difference between the infliximab and placebo groups in MCS was not statistically significant.

**Figure 4:**
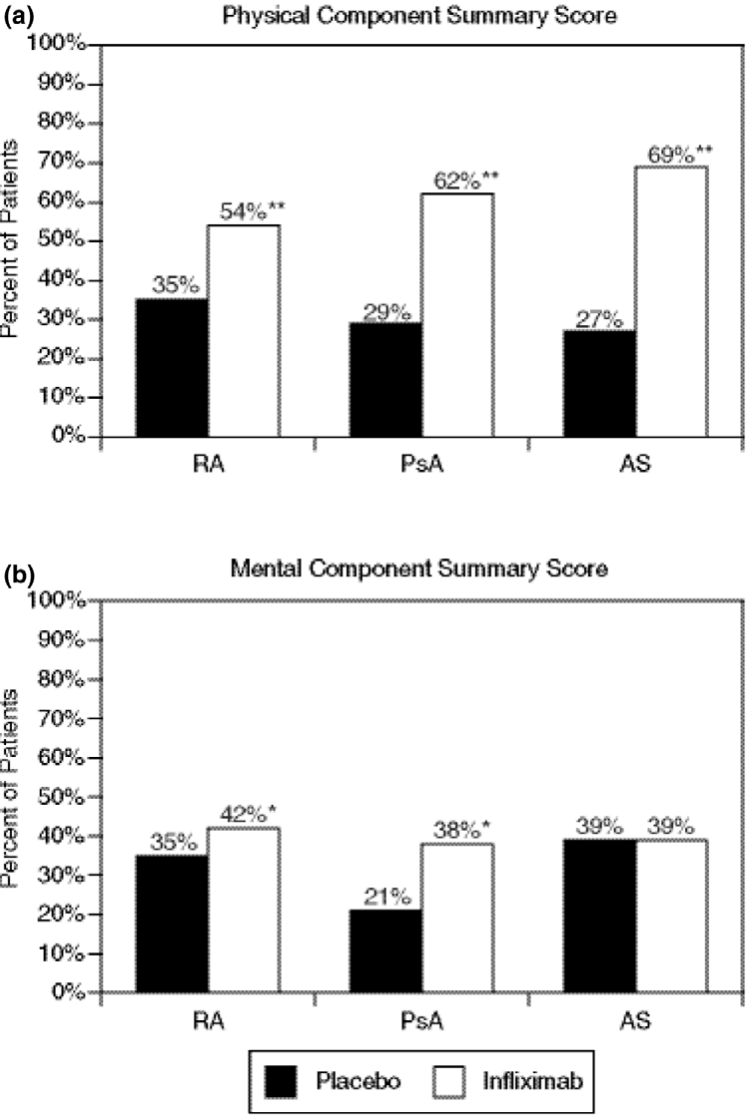
**Clinically meaningful improvement in health-related quality of life**. Percentage of patients who achieved a clinically meaningful improvement (≥5 points) from baseline to the first assessment time point (rheumatoid arthritis (RA): week 6 or 10, psoriatic arthritis (PsA): week 14, and ankylosing spondylitis (AS): week 12) in the physical (a) or mental (b) component summary scores of the SF-36.

Compared with patients who received placebo, significantly more patients who were treated with infliximab achieved a clinically meaningful improvement (≥5 points) [13] in PCS and MCS scores from baseline to the first assessment time point (Figure 4).

### Persistence of improvement in quality of life

The initial improvement in physical HRQoL was either maintained or continuously slightly improved over time thereafter. Among patients with RA who received infliximab, the change in PCS from baseline to week 54 was 10.1, compared with 4.4 for the placebo group (*P *< 0.01). Changes from baseline to week 54 (3.2 for infliximab versus 3.4 for placebo) in MCS were similar between the treatment groups (*P *> 0.05). Among patients with AS, the change from baseline to week 24 in PCS was 9.9 in the infliximab group compared with 0.7 in the placebo group (*P *< 0.01). There was no significant difference between the groups in the change from baseline to week 24 in MCS (3.3 for infliximab versus 2.7 for placebo, *P *> 0.05). The improvement in PCS scores among patients treated with infliximab was maintained through 102 weeks in AS patients, and through 54 weeks in patients with PsA and those with RA.

## Discussion

The burden of RA, PsA, and AS on patients, their caregivers, and society as a whole is significant. Chronic inflammation leads to pain, stiffness, joint erosions, ankylosing features, and impaired mobility, which can result in functional disability, increased health care costs, and reduced employability. These factors and the strategies patients use to cope with disease burden contribute to an impaired HRQoL for affected patients. In this study, we directly compared the HRQoL of patients with three inflammatory rheumatic diseases. The distribution of baseline PCS scores for all three diseases were shifted to the left of the distribution for the general United States population. These results demonstrate that these patients had greatly impaired physical HRQoL relative to the general population.

The data used in this analysis were from patients who participated in clinical trials of the anti-tumor necrosis factor-α (TNFα) agent infliximab. To be eligible for these studies, patients must have had moderate-to-severe disease activity at baseline. The comparison of the mean baseline PCS, as well as scores for the individual scales, showed that patients with PsA and those with AS had similarly impaired HRQoL relative to patients with RA. This finding is different from the reports of previous studies in which patients with RA had lower physical SF-36 summary scores compared with those with AS [3] or PsA [4].

One potential explanation for this discrepancy is the disease severity of patients in each of the cohorts. In the Toronto study that compared the quality of life of patients with RA to that of patients with PsA [4], the mean number of active joints in patients with PsA was 6, while the baseline mean number of tender and swollen joints for patients in the IMPACT II study was 25 and 14 joints, respectively [10]. Similarly, the mean number of active joints in patients with RA in the Toronto study was lower (6) than the mean number of tender and swollen joints for patients in ATTRACT (32 and 22 joints, respectively) and START (24 and 17 joints, respectively). In the analysis of patients with RA or AS in a Dutch study [3], the mean baseline Bath Ankylosing Spondylitis Disease Activity score was 3.9 compared with a mean score of 6.4 for patients in the ASSERT trial [9]. Disease activity assessments for patients with RA in the Dutch study were different from those used in ATTRACT or START studies; however, the mean baseline SF-36 physical component summary score in the RA cohort of the present study (29) was lower than those of patients with RA in the Dutch study (35.7 in men and 34.3 in women).

Results of the present analysis also demonstrate the significant improvement in HRQoL after treatment with the TNFα inhibitor infliximab. After adjustment for age, sex, and disease duration, patients in all the disease cohorts who received infliximab demonstrated significantly greater improvement from baseline in PCS than those who received placebo. Role physical and bodily pain were the domains that had the greatest magnitude of change in each disease cohort. Early improvement was evident at the first assessment point, which ranged from week 6 to week 14 depending on the study design. The magnitude of the difference in the change from baseline in PCS between the infliximab and placebo groups was comparable among the three diseases.

Patients with RA and PsA treated with infliximab also showed greater improvement in MCS than those treated with placebo, although the improvement in MCS between infliximab and placebo groups was not statistically significant in the AS cohort.

Improvements in PCS persisted throughout the placebo-controlled period of each study. The longest placebo-controlled period was in the ATTRACT study, in which improvements in mean PCS were sustained and increased slightly through week 54.

We observed several notable differences in the changes in the placebo group across diseases. Changes in PCS in the placebo group were lower in the PsA and AS cohorts compared with the RA cohort. All patients in the RA cohort, including those in the placebo group, were incomplete responders to MTX at baseline and received concomitant MTX throughout the study. However, eligibility for the trials was determined using clinical signs and symptoms criteria, not quality of life measurements.

There are some potential limitations of this retrospective study, and the results should be interpreted with caution. Although the SF-36 is robust for comparisons across diseases [14], each study assessed quality of life at different time points according to the study design. Thus, the first quality of life assessment ranged from week 6 in START to week 14 in IMPACT II. Moreover, each study had different selection criteria, study design, and infliximab dosages, making comparisons of HRQoL difficult. Although all 3 rheumatic diseases evaluated were immune-mediated inflammatory disorders that share some pathophysiological characteristics, comparisons of patients with different diseases should always be interpreted with caution. In the analysis, we adjusted SF-36 scores for age, sex, and disease duration, to attempt to control for potentially confounding factors.

## Conclusion

Patients with rheumatic disease have an impaired physical HRQoL relative to the general population, and the magnitude of that impairment is similar among patients with moderate-to-severe RA, PsA, or AS who were included in clinical trials with infliximab. Treatment with infliximab resulted in significantly improved HRQoL.

## Abbreviations

ANCOVA = analysis of covariance; AS = ankylosing spondylitis; ASSERT = Ankylosing Spondylitis Study for the Evaluation of Recombinant Infliximab Therapy; ATTRACT = Anti-TNF Trial in Rheumatoid Arthritis with Concomitant Therapy; CRP = C-reactive protein; HRQoL = health-related quality of life; IMPACT II = Infliximab Multinational Psoriatic Arthritis Clinical Trial II; MCS = mental component summary score; MTX = methotrexate; PsA = psoriatic arthritis; PCS = physical component summary score; RA = rheumatoid arthritis; SF-36 = Short Form Health Survey-36; START = Safety Trial for Rheumatoid Arthritis with REMICADE Therapy; TNF = tumor necrosis factor.

## Competing interests

JS, AK, DH, JB, and RW have received research funding and/or consulting fees from Centocor. CH, NZ, MR, DB, and MB are employees of Centocor and own Johnson & Johnson stock.

## Authors' contributions

CH designed analysis with input from the rest of the authors. CH and NZ conducted the analysis. All authors interpreted the data. CH wrote the manuscript with the assistance of a medical writer. All authors critically reviewed the manuscript and approved of the final manuscript before submission.
